# Impact of electronic and blended learning programs for manual perineal support on incidence of obstetric anal sphincter injuries: a prospective interventional study

**DOI:** 10.1186/s12909-018-1363-3

**Published:** 2018-11-12

**Authors:** Hadil Ali-Masri, Sahar Hassan, Erik Fosse, Kaled M. Zimmo, Mohammed Zimmo, Khaled M. K. Ismail, Åse Vikanes, Katariina Laine

**Affiliations:** 1 Department of Obstetrics, Palestine Medical Complex, Ramallah, Palestine; 2 The Intervention Centre, Oslo University Hospital, Rikshospitalet, Oslo, Norway; 3 Institute of Clinical Medicine, Faculty of Medicine, University of Oslo, Oslo, Norway; 4 Faculty of Pharmacy, Nursing and Health Professions, Birzeit University, Ramallah, Palestine; 5 Department of Obstetrics, Al Aqsa Martyrs Hospital, Gaza, Palestine; 6 Department of Obstetrics, Al Shifa Hospital, Gaza, Palestine; 7 Department Obstetrics and Gynaecology, Ain Shams University, Cairo, Egypt; 8 Department of Obstetrics, Oslo University Hospital, Ullevål, Oslo, Norway; 9 Department of Health Management and Health Economics, Institute for Health and Society, University of Oslo, Oslo, Norway

**Keywords:** Animation, Blended, Face-to-face, manual, OASIS, Palestine, Perineum, Support, Training

## Abstract

**Background:**

Obstetric anal sphincter injuries (OASIS) are associated with anal incontinence, dyspareunia and perineal pain. Bimanual perineal support technique (bPST) prevents OASIS. The aim of this study was to assess the effect of two different bPST training-methods on OASIS incidence.

**Methods:**

This is a prospective-interventional quality improvement study conducted in two Palestinian maternity units between June 1 2015 and December 31 2016. Women having spontaneous or operative vaginal-delivery at ≥24 gestational-weeks or a birthweight of ≥1000 g (*n* = 1694) were recruited and examined vaginally and rectally immediately after vaginal birth by a trained assessor. Data on baseline OASIS incidence were collected during Phase-1 of the study. Subsequently, birth attendants in both maternity units were trained in bPST using two training modalities. A self-directed electronic-learning (e-learning) using an animated video was launched in phase-2 followed by a blended learning method (the animated e-learning video+ structured face-to-face training) in phase-3. OASIS incidence was monitored during phases-2 and 3. Variations in OASIS incidence between the three phases were assessed using Pearson-χ^2^-test (or Fisher’s-Exact-test). The impact of each training-method on OASIS incidence was assessed using logistic-regression analysis.

**Results:**

A total of 1694 women were included; 376 in phase-1, 626 in phase-2 and 692 in phase-3. Compared to Phase-1, OASIS incidence was reduced by 45% (12.2 to 6.7%, aOR: 0.56, CI; 0.35–0.91, *p* = 0.018) and 74% (12.2 to 3.2%, aOR, 0.29, CI; 0.17–0.50, *p* < 0.001) in phases-2 and 3, respectively. There was also a significant reduction in OASIS incidence by 52% from phase-2 to phase-3 (6.7% (42/626) to 3.2% (22/692), *p* = 0.003).

These reductions reached statistical significance among parous-women only (aOR: 0.18, CI; 0.07–0.49, *p* = 0.001) after the first training method tested in phase-2. However, the reduction was significant among both primiparous (aOR: 0.39, CI; 0.21–0.74, *p* = 0.004) and parous-women (aOR: 0.11, CI; 0.04–0.32, p < 0.001) after implementing the blended learning method in phase-3.

**Conclusion:**

The animated e-learning video had a positive impact on reducing OASIS incidence. However, this reduction was enhanced by the use of a blended learning program combining both e- learning and face-to-face training modalities.

**Study registration number:**

ClinicalTrialo.gov identifier: NCT02427854, date: 28 April 2015.

**Electronic supplementary material:**

The online version of this article (10.1186/s12909-018-1363-3) contains supplementary material, which is available to authorized users.

## Background

Obstetric anal sphincter injuries (OASIS) are the leading cause of dyspareunia, perineal pain and female anal incontinence [1–3]. Recognition and suturing of OASIS require well-trained and experienced clinicians [4, 5]. Despite primary repair, between 15 to 61% of women who sustain OASIS still develop anal incontinence [2]. Furthermore, there is five times increased risk of recurrence of OASIS in a subsequent vaginal delivery [6]. In view of this, there have been several efforts to reduce OASIS occurrence by modifying intrapartum predisposing factors such as using vacuum instead of forceps and mediolateral instead of midline cut when episiotomy is indicated [5]. However, primiparity and macrosomia are among the prominent risk factors of OASIS, which cannot be modified [7, 8]. Several studies have shown that protecting the perineum with the bimanual perineum support technique (bPST) or the “Finnish grip” plays an effective role in reducing OASIS incidence even in high risk births [9–11].

Mobile health education (m-health) has emerged as a fast and low-cost e-learning route, providing solutions to the challenges of training of health workers, particularly, in limited-resource settings [12]. More than 80% of the population living in rural areas has access to mobile devices [13], which seem to contribute to the improvement of healthcare including maternal healthcare, in many low-middle-income countries [14]. A recent systematic review has demonstrated the success of different m-health interventions undertaken in Sub-Saharan Africa, East and South Asia and in the Middle East, in improving the quality of antenatal and postnatal care, increasing awareness and knowledge of pregnant women, tracking vital signs and obstetric emergencies and also collecting pregnancy related data [15].

Blended-learning, which refers to a structured incorporation of electronic and face-to-face learning [16], has been shown to be an effective modality of training in different medical fields [17–19].

The main aim of this study was to assess the impact of two different training-methods on OASIS incidence; a stand-alone animated e-learning video providing practical instructions about bPST, and a blended learning modality involving the e-learning video as well as structured face-to-face training.

## Methods

The reporting of this prospective quality-improvement interventional study followed the Standards for Quality Improvement Reporting Excellence (SQUIRE) 2.0 statement for quality-improvement studies. The study was conducted over 14 months, in the period between June 1st 2015 and December 31st 2016, within two governmental maternity units in Palestine. One maternity unit is located in the West Bank and the other in Gaza, with an average birth rate of 8000–10,000 births per annum. The maternity unit in the West Bank is qualified as a training center for intern and resident doctors, medical students and midwifery students, while the Gaza unit does not provide similar training programs. In the participating maternity units, doctors are involved in high risk and instrumental deliveries while midwives manage the low risk deliveries. Episiotomy and perineal tears are usually assessed and repaired by doctors only. Vacuum is the only method used for operative vaginal delivery and right mediolateral episiotomy is the recommended episiotomy technique in both maternity units. Perineal protection is provided during crowning of the baby’s head by placing the dominant hand flat on the posterior perineum while the other hand is used to protect the urethra. The mother is instructed to push with contractions only. Although this could be considered a form of bimanual perineal support, there are several inherent differences from bPST [9] where the non-dominant hand controls the speed of the delivery of the baby’s head while the dominant hand supports the perineum in a way resembling a grip as the thumb and index fingers are placed on the lateral sides of the posterior perineum to squeeze towards the midline and the flexed middle finger supports the perineal body. Meanwhile, the need for an episiotomy is evaluated. Just before the delivery of the baby’s head, the mother is instructed to stop pushing and wait for the uterine contractions to spontaneously deliver the head to avoid its sudden expulsion. Following delivery of the head, perineal support is continued during delivery of the shoulders (see Additional file 1). Women in this study did not receive antepartum training or education about the bPST. The technique and instructions were to be explained to the mother after admission just before the second stage of labor was approached.

We used two training-methods on two consecutive time periods; self-directed animated e-learning video and blended-learning (a combination of the animated e-learning video and the traditional face-to-face training).

### E-learning training package (training-method 1)

A four-minute e-learning video© (Additional file 1) was developed for this study. The video contains an introduction about obstetric perineal trauma and animated demonstration of bPST with voiceover in Arabic and English. Each maternity unit received two tablets with both video versions. These were wall-mounted in labor ward and staff meeting rooms of each of the participating units simultaneously. Birth attendants (doctors and midwives) were encouraged to watch the video as many times as they want, nevertheless, no assistance or explanations were provided.

### Blended-learning training package (training-method 2)

This included the previously described animated e-learning video and face-to-face training. The tablets were kept on site during and after the face-to-face training. One senior obstetrician (author KL) and two tutor midwives from Oslo University Hospital conducted the face-to-face training with the assistance of three local doctor research fellows (authors HAM, KZ and MZ) during which more than 90% (84 doctors and midwives) of birth attendants were trained. The structured face-to-face training consisted of an initial workshop run in two successive days in each of the two maternity units, to allow as many birth attendants as possible to be trained, where those who were working on the first day were able to attend in the second day and vice versa. The workshop included lectures on OASIS rates, diagnosis, risk factors and prevention, illustration of bPST and accurate technique of episiotomy when indicated followed by instructor-guided practical training on birthing simulators. The workshop was followed by a 10 day period of supervised real-life assessment and training in the labor rooms by the Norwegian tutor midwives; one midwife within each of the two maternity units.

### Study population

The study population was selected randomly, reducing the risk of selection bias. Women were considered eligible for inclusion if they had a vaginal birth, were ≥ 24 gestational weeks or had a baby with a birth weight ≥ 1000 g, provided verbal consent to be examined and there was a trained assessor available on labor ward. All examinations were performed by one of the three research fellows (authors HAM, KZ and MZ), or one of four doctors working in the participating units, who were previously thoroughly trained in postnatal perineal assessment by the three research fellows [20]. The seven assessors were equally distributed throughout the study period; four assessors undertook the examinations in Gaza maternity unit and three assessors in the West Bank maternity unit. The trained assessors were not involved in the actual birth and three of them had pre-scheduled working hours while the other four were available at random times that the birth attendants could not predict. The assessment was only clinical involving both vaginal and rectal examinations, which took place during morning, evening or night shifts. The assessors were not blinded to whether the birth attendants had been trained or not after implementation of training-method 2 but not after training-method 1, as it was not possible to register and identify the birth attendants who had used the animated e-learning method.

The main outcome measure was OASIS incidence calculated as percent number of OASIS cases per number of total women examined in each of the study phases. The definition and classification of OASIS were based on the diagnoses O70.2 and O70.3 in the ICD-10 system [21] as shown in Table 1.

**Table 1:** ICD-10 classification of perineal injuries

Degree	Definition
First	Injury to vaginal mucosa or perineal skin only
Second	Injury to vaginal mucosa or perineal skin and superficial perineal muscles
Third	Injury to anal sphincter muscles and subdivided into
3A	< 50% of external anal sphincter muscles is injured
3B	> 50% of external anal sphincter muscle is injured
3C	External and internal anal sphincter muscles are injured
Fourth	Injury involving external and internal anal sphincter muscles and anal epithelium

**Fig. 1:**
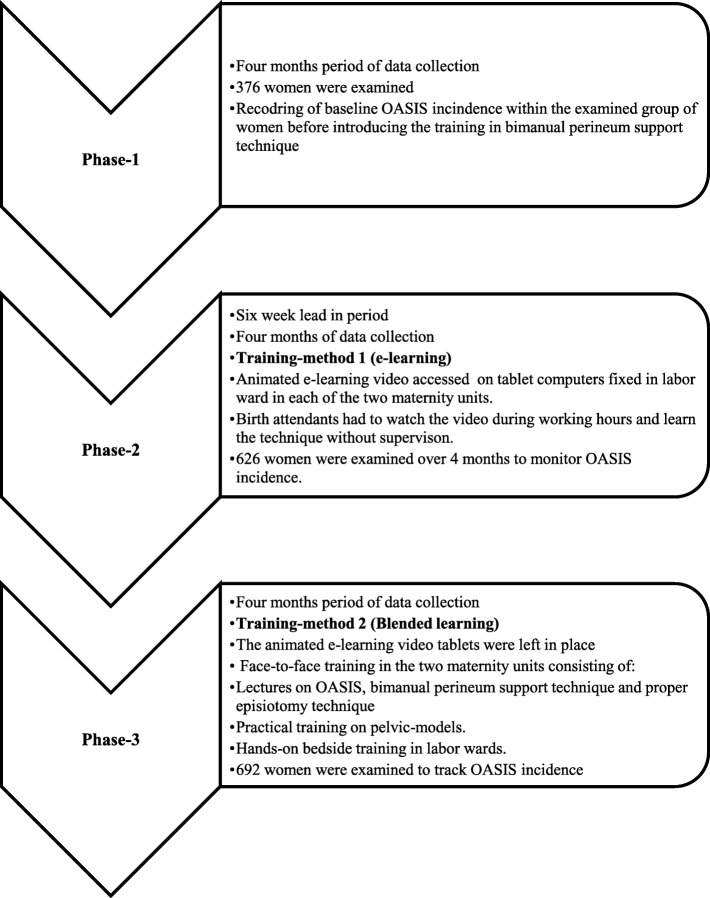
Flow chart presenting the study phases and the training-intervention timeline

The study was designed to run in three consecutive phases (Fig. 1).

In phase-1, 376 women were examined over four-month period prior to any bPST training interventions for baseline data on OASIS incidence.

In phase-2, 626 women were examined over a four-month period after the first training-intervention (animated e-learning video); to monitor possible effects on OASIS incidence.

In phase-3, 692 women were examined over a four month period after introducing the blended learning method (animated e-learning video and additional face-to-face training); in order to track the impact of the interventions on OASIS incidence.

### Data collection

This study is a sub-study of a population based birth cohort study “Palestinian Perineum and Birth Complications Study”. Data collection was previously described by Sahar et al. [22]. Birth attendants collected data prospectively during the three study phases, using paper and electronic case registration forms, identified by serial numbers. All data were then transferred to Service (Tjenester) for Sensitive Data (TSD) platform at University of Oslo (tsd-drift@usit.uio.no). The TSD platform is used by researchers working at the university and in other public research institutions to collect, store, analyze, and share sensitive data in compliance with the Norwegian regulations regarding individuals’ privacy. Serial numbers designated to women, examined in this study, were obtained and subsequently used to retrieve their data which were de-identified at this stage.

### Statistics

Based on data collected for baseline in phase-1, statistical power calculations were conducted, showing that at least 355 women were required in each study phase to demonstrate a 50% reduction in OASIS incidence after the training-interventions, given a statistical power of 80 and 95% confidence interval.

Categorical data were presented as frequencies and continuous data as means and standard deviations (SD). Body mass index (BMI), birthweight and duration of second stage of labor were categorized. Variations among the three study phases were assessed using One-Way-ANOVA-test for continuous and Pearson-χ^2^ or Fisher’s-Exact tests (if cell counts < 5) for categorical variables. Univariate analysis by χ^2^-test was performed to explore risk factors associated with OASIS. All risk factors with *p* value < 0.2 were used in the multiple logistic regression analysis to determine which factors were independently associated with OASIS in each phase. Accordingly, those significant risk factors were adjusted for using multiple regression analysis to determine the direct impact of the two training-methods on OASIS incidence and results were presented by adjusted OR (aOR) for OASIS with 95% CI. The same analysis was repeated stratified according to parity. The significance level was set at p value < 5%. The analyses were conducted using SPSS version 24.0 (IBM, Armonk, NY, USA).

## Results

A total of 1694 women were included, among those 22.2% (*n* = 376) were examined before the training-intervention (phase-1), 36.9% (*n* = 626) during phase-2 and 40.9% (*n* = 692) during phase-3.

Clinical characteristics and perineal status in the three study phases are presented in Table 2.

**Table 2:** Clinical characteristics and perineal status of study population during the three study phases

Characteristics	Phase-1 (*N* = 376)	Phase-2 (N = 626)	Phase-3 (N = 692)	P^1^
Age (years)	25 ± 5.0	26 ± 5.6	27 ± 5.7	< 0.001
Gestational age	39.0 ± 1.6	39.0 ± 1.8	39 ± 1.8	0.597
BMI^2^				
< 18.5	–	–	–	
18.5–24.99	66 (17.6)	116 (18.5)	129 (18.6)	0.887
25–29.99	198 (52.7)	313 (50.0)	346 (50.0)	
≥ 30	105 (27.9)	190 (30.4)	210 (30.3)	
Missing	7 (1.9)	7 (1.1)	7 (1.0)	
Primiparous women	195 (51.9)	279 (44.6)	256 (37.0)	< 0.001
Parous women	181 (48.1)	347 (55.4)	436 (63.0)	
Method of vaginal birth				
Spontaneous	363 (96.5)	587 (93.8)	671 (97.0)	0.011
Vacuum	13 (3.5)	39 (6.2)	21 (3.0)	
Fetal presentation				
Cephalic	373 (99.2)	619 (98.9)	685 (99.0)	0.663
Breech	2 (0.5)	7 (1.1)	6 (0.9)	
Occiput posterior	1 (0.3)	0 (0.0)	1 (0.1)	
Onset of labor				
Spontaneous	328 (87.2)	513 (82.0)	605 (87.4)	0.010
Induced	48 (12.8)	113 (18.0)	87 (12.6)	
Second stage duration (minutes)				
< 30	256 (68.1)	332 (53.0)	432 (62.4)	< 0.001
30–59	82 (21.8)	150 (24.0)	161 (23.3)	
≥ 60	22 (5.9)	81 (12.9)	64 (9.2)	
Missing	16 (4.3)	63 (10.1)	35 (5.1)	
Birthweight (grams)				
< 3000	86 (22.9)	170 (27.2)	174 (25.1)	0.848
3000–3499	172 (45.7)	269 (43.0)	309 (44.7)	
3500–3999	93 (24.7)	145 (23.2)	167 (24.1)	
≥ 4000	25 (6.6)	42 (6.7)	42 (6.1)	
Intact Perineum	152 (40.4)	325 (52.0)	426 (61.6)	< 0.001
Episiotomy	134 (35.6)	166 (26.5)	124 (18.0)	< 0.001
First degree tear	42 (11.2)	85 (13.6)	93 (13.4)	0.492
Second degree tear	54 (14.4)	72 (11.5)	98 (14.2)	0.276
OASIS^3^	46 (12.2)	42 (6.7)	22 (3.2)	< 0.001

• Categorical data were presented by n/N (%), and continuous variables by mean ± SD

• P^1^; differences were assessed by One-Way-ANOVA test for continuous variables and Pearson-χ^2^ test or Fisher’s Exact test (for cells with counts< 5) for categorical variables.

• BMI^2^; body mass index = maternal weight in kilograms/(height in meters)^2^

• OASIS^3^; obstetric anal sphincter injuries.

There were significant variations between the study phases in proportions of primiparous women (37.0–51.9%), vacuum assisted delivery (3.0–6.2%), induced labor (12.6–18.0%) and duration of second stage of labor ≥60 (5.9–12.9%).

The OASIS incidence was reduced by 45% from phase-1 to phase-2 (12.2% (46/376) to 6.7% (42/626), *p* = 0.004), by 52% from phase-2 to phase-3 (6.7% (42/626) to 3.2% (22/692), *p* = 0.003) and by 74% from phase-1 to phase-3 (12.2% (46/376) to 3.2% (22/692), *p* < 0.001). There was also a significant reduction in episiotomy rates and a significant increase of intact perineum proportions after implementing training-method 1 and 2 (Table 1).

Primiparity, birthweight ≥4000 g and duration second stage ≥60 min were associated with increased risk of OASIS. When applying multiple regression analysis, adjustment for parity, birthweight and duration of second stage did not change the effect of the two training-interventions on reducing OASIS incidence; training-method 1 (aOR: 0.56, CI; 0.35–0.91, *p* = 0.018) and training-method 2 (aOR: 0.29, CI; 0.17–0.50, *p* < 0.001).

### Primiparous women

Compared to phase-1, phases-2 and 3 had significantly larger proportions of women with prolonged second stage of labor ≥60 min (25.8 and 20.7% vs 11.3%) and intact perineum (25.4 and 34.8% vs 18.0%) and lower episiotomy rates (52.7 and 39.5% vs 64.6%) as shown in Table 3.

**Table 3:** Clinical characteristics and perineal status among primiparous women during the three study phases

Characteristics	Phase-1 (*N* = 195)	P^1^	Phase-2 (*N* = 279)	P^2^	Phase-3 (*N* = 256)	P^3^
Age (years)	23 ± 4.0	0.040	24 ± 5.0	0.328	23 ± 5.0	0.306
Gestational age (weeks)	39 ± 1.5	0.333	39 ± 1.6	0.099	39 ± 2.0	0.022
BMI^4^						
< 18.5	–		–		–	
18.5–24.99	46 (23.6)	0.543	67 (24.0)	0.171	49 (19.1)	0.471
25–29.99	99 (50.8)		131 (47.0)		141 (55.1)	
≥ 30	45 (23.1)		77 (27.6)		64 (25.0)	
Missing	5 (2.6)		4 (1.4)		2 (0.8)	
Method of vaginal birth						
Spontaneous	184 (94.4)	0.166	253 (90.7)	0.146	241 (94.1)	> 0.999
Vacuum	11 (5.6)		26 (9.3)		15 (5.9)	
Fetal presentation						
Cephalic	193 (99.0)	> 0.999	277 (99.3)	0.433	252 (98.4)	0.703
Breech	1 (0.5)	> 0.999	2 (0.7)	0.433	4 (1.6)	0.395
Occiput posterior	1 (0.5)	0.411	–	0.433	–	0.432
Onset of labor						
Spontaneous	161 (82.6)	0.447	222 (79.6)	0.040	221 (86.3)	0.292
Induced	34 (17.4)		57 (20.4)		35 (13.7)	
Second stage duration (minutes)						
< 30	104 (53.3)	< 0.001	94 (33.7)	0.174	105 (41.0)	0.009
30–59	62 (31.8)		78 (28.0)		81 (31.6)	
≥ 60	22 (11.3)		72 (25.8)		53 (20.7)	
Missing	7 (3.6)		35 (12.5)		17 (6.6)	
Birthweight (grams)						
< 3000	53 (27.2)	0.317	93 (33.3)	0.161	84 (32.8)	0.102
3000–4399	85 (43.6)		118 (42.3)		121 (47.3)	
3500–3999	47 (24.1)		51 (18.3)		45 (17.6)	
≥ 4000	10 (5.1)		17 (6.1)		6 (2.3)	
Intact Perineum	35 (18.0)	0.057	71 (25.4)	0.023	89 (34.8)	< 0.001
Episiotomy	126 (64.6)	0.011	147 (52.7)	0.002	101 (39.5)	< 0.001
First degree tear	17 (8.7)	0.751	27 (9.7)	0.334	32 (12.5)	0.224
Second degree tear	31 (15.9)	0.396	53 (19.0)	0.018	71 (27.7)	0.003
OASIS^5^	31 (15.9)	0.346	35 (12.5)	0.028	17 (6.6)	0.002

• Differences were assessed for each pair of the study phases

• Categorical data were presented by n/N (%), and continuous variables by mean ± SD

• Differences were assessed by independent t-test for continuous variables and Pearson- Pearson-χ^2^ test or Fisher’s Exact test (for cells with counts< 5) for categorical variables.

• P^1^; difference between phase-1 and phase-2, P^2^; difference between phase-2 and phase-3, P^3^; difference between phase-1 and phase-3.

• BMI^4^; body mass index = maternal weight in kilograms/(height in meters)^2^

• OASIS^5^; obstetric anal sphincter injuries.

Vacuum assisted delivery and duration second stage of labor ≥60 min were associated with risk of OASIS in phase-1. No significant risk factors were identified in phase-2 or 3. Episiotomy, induction of labor, BMI, fetal presentation and birthweight were not associated with OASIS. Although OASIS incidence was reduced following training-method 1 (12.5% (35/279) vs 15.9% (31/195)), this reduction was not statistically significant after adjusting for the identified significant risk factors associated with OASIS (vacuum assisted delivery and duration second stage of labor ≥60 min); (aOR:0.81, CI; 0.47–1.4, *p* = 0.465). In contrast, the training-method 2 was associated with a significant reduction in OASIS incidence (6.6% (17/256) vs. 15.9% (31/195), aOR: 0.39, CI; 0.21–0.74, *P* = 0.004).

There was an increase in the incidence of second degree tears during phase-3 which was independently associated with training-method 2 (aOR: 2.3, CI; 1.4–3.7, *p* = 0.002) and episiotomy use (aOR:1.5, CI; 1.0–2.3, *p* = 0.033).

### Parous women

Table 4 shows clinical characteristics and perineal status among parous women before and after the training-interventions. OASIS incidence was significantly reduced from 8.3% (15/181) in phase-1 to 2.0% (7/347) in phase-2 down to 1.1% (5/436) in phase-3. No significant variation in episiotomy rate was observed, but proportions of intact perineum were significantly higher and incidence of second degree tear were significantly lower after the two training methods compared to before (Table 3).

**Table 4:** Clinical characteristics and perineal status among parous women during the three study phases

Characteristics	Phase-1 (*N* = 181)	P^1^	Phase-2 (*N* = 347)	P^2^	Phase-3 (*N* = 436)	P^3^
Age (years)	27 ± 5.0	0.012	28 ± 5.0	0.900	28 ± 5.0	0.008
Gestational age (weeks)	39 ± 1.7	0.893	39 ± 2.0	0.519	39 ± 2.0	0.683
BMI^4^						
< 18.5	–		–		–	
18.5–24.99	20 (11.0)		49 (14.1)		80 (18.3)	
25–29.99	99 (54.7)	0.617	182 (52.4)	0.194	205 (47.0)	0.056
≥ 30	60 (33.1)		113 (32.6)		146 (33.5)	
Missing	2 (1.1)		3 (0.9)		5 (1.1)	
Method of birth						
Spontaneous	179 (98.9)	0.101	334 (96.3)	0.037	430 (98.6)	> 0.999
Vacuum	2 (1.1)		13 (3.7)		6 (1.4)	
Fetal presentations						
Cephalic	180 (99.4)		342 (98.6)	0.477	433 (99.3)	
Breech	1 (0.6)	0.440	5 (1.4)	0.251	2 (0.5)	> 0.999
Occiput posterior	–		–	> 0.999	1 (0.2)	
Onset of labor						
Spontaneous	167 (92.3)	0.010	291 (83.9)	0.096	384 (88.1)	0.152
Induced	14 (7.7)		56 (16.1)		52 (11.9)	
Second stage duration (minutes)						
< 30	152 (84.0)	0.001	238 (68.6)	0.505	327 (75.0)	0.006
30–59	20 (11.0)		72 (20.7)		80 (18.3)	
≥ 60	–		9 (2.6)		11 (2.5)	
Missing	9 (5.0)		28 (8.1)		18 (4.1)	
Birthweight						
< 3000	33 (18.2)	0.626	77 (22.2)	0.907	90 (20.6)	0.709
3000–3499	87 (48.1)		151 (43.5)		188 (43.1)	
3500–3999	46 (25.4)		94 (27.1)		122 (28.0)	
≥ 4000	15 (8.3)		25 (7.2)		36 (8.3)	
Intact Perineum	117 (64.6)	0.045	254 (73.2)	0.210	337 (77.3)	0.001
Episiotomy	8 (4.4)	0.681	19 (5.5)	> 0.999	23 (5.3)	0.694
First degree tear	25 (13.8)	0.450	58 (16.7)	0.317	61 (14.0)	> 0.999
Second degree tear	23 (12.7)	0.004	19 (5.5)	0.760	27 (6.2)	0.009
OASIS^5^	15 (8.3)	0.001	7 (2.0)	0.387	5 (1.1)	< 0.001

• Differences were assessed between each pair of the study phases

• Categorical data were presented by n/N (%), and continuous variables by mean ± SD

• Differences were assessed by independent t-test for continuous variables and Pearson- Pearson-χ^2^ test or Fisher’s Exact test (for cells with counts< 5) for categorical variables

• P^1^; difference between phase-1 and phase-2, P^2^; difference between phase-2 and phase-3, P^3^; difference between phase-1 and phase-3

• BMI^4^; body mass index = maternal weight in kilograms/(height in meters)^2^

• OASIS^5^; obstetric anal sphincter injuries

Adjusted risk factors for OASIS were episiotomy, BMI and duration of second stage of labor. Episiotomy was the only factor associated with an increased risk of OASIS (aOR: 3.8, CI; 1.2–12.1, *p* = 0.025). Both training-methods had an effect on the odds for OASIS; training-method 1 (aOR: 0.18, CI; 0.07–0.49, *p* = 0.001) and training-method 2 (aOR: 0.11, CI; 0.04–0.32, *p* < 0.001).

## Discussion

This study has shown a significant reduction in OASIS incidence after introducing bPST using animated e-learning video alone or combined with face-to-face training. The blended learning methodology reduced OASIS incidence by two thirds, and was almost twice as effective as the e-learning video alone. Moreover, while the impact of the blended learning was statistically significant among primiparous and parous women, the effect of the animated video alone was only significant among parous women.

Changes in the clinical characteristics and risk factors between the three study phases do not explain the rapid reduction in OASIS incidence.

Non-blinded clinical studies could influence clinical practice. However, the transient increase in the rate of the operative delivery during phase-2 is believed to be random and independent of the training interventions. The two training methods were specifically focused and hence are not believed to have influenced other intrapartum management protocols throughout the study period.

Nilsson et al. [23], demonstrated that video-training in the management of postpartum hemorrhage was as effective as hands-on training in a secondary health care in Kenya. However, those who participated in that study were senior nursing students who were more likely to be familiar with postpartum hemorrhage management as part of their curriculum. In contrast, birth attendants in our setting were not aware of bPST prior to this study. Training-method 1 required the birth attendants to learn directly from the animated e-learning video without a supervisor. Hence, the supervised training and interactive discussions related to problems encountered during the use of the technique are not possible using the animated video alone. The combination of e-learning and traditional face-to-face training has become popular enabling integration of theoretical knowledge and clinical practice [24].

There are several plausible explanations for the differences in the effects of the two tested training methods. We have previously shown that the animated e-learning video has been approved as a clear and comprehensible tool feasible to use as an independent training method [25]. It is possible that birth attendants did not get the opportunity to see the video repeatedly until they feel competent to use bPST, which is necessary for this tool to achieve its maximum effect. Indeed, frequent exposure to the same instructional material has been found to be associated with better learning outcomes [26], but the work overload in our clinical setting may not have made watching the video frequently possible. Clinicians often utilize their smart mobile devices for professional tasks [27], hence making the training video available on this platform could enhance its dissemination by enabling birth attendants to watch the video at their convenience [28]. Secondly, it is well documented that changing clinical practice is often faced by resistance and delayed adoption [29]. It is possible that initially, birth attendants were not fully engaged in the animation-based training since they had no prior education about the clinical effectiveness of bPST, something that is believed to hinder its use [25]. The blended learning modality enabled birth attendants to see and speak to experts in the field, which could have bridged the engagement gap and resulted in a substantial reduction in OASIS incidence during phase-3. In support of our study findings is the reporting of a recent systematic review that blended learning is equally or more effective than self-directed e-learning [30]. However, in that review, the effect of the different learning modalities focused on improving health professionals’ knowledge rather than evaluating their clinical competencies. Although there is some evidence of clinical skills’ improvement as a result of engaging blended learning [24], the variation in clinical contexts and the diversity of learning approaches highlight the need for more concrete research on the role of blended learning in the actual clinical scenarios [24].

There is still debate about the role of hands-on support of the perineum in the prevention of severe obstetric trauma. A recent update of a Cochrane systemic review has reported that the hands-on perineum support has no effect on OASIS risk compared to the hands-off method [31]. Nevertheless, the low quality of the included studies and the heterogeneity of the studied perineal support techniques did not provide robust evidence. Our findings, however, are in line with earlier studies’ reporting that the bPST is effective at reducing severe perineal trauma [9–11, 32–34]. Implementation of the bPST has resulted in more than 50% reduction of OASIS incidence from 4 to 1.9% in Norway and from 7 to 3.4% in Denmark, respectively [9, 11]. In the UK, the incidence of major OASIS (third degree c and fourth degree tears) was significantly reduced after the use of bPST [10].

It has been reported that the hands-on technique is associated with higher episiotomy rate than the hands-off technique [30]. In this study, we observed a marked reduction of episiotomy rate among primiparous women after implementing the bPST. One explanation may be that both training-interventions in this study included teaching that episiotomy should be done on indication only, which is particularly important for primiparous women in Palestine who are often being cut. In addition, we observed an increase in the incidence of spontaneous second degree tears after the training-intervention among primiparous women which was independent of the concomitant reduction in episiotomy rate, but could be argued to be due to the reduced severity of perineal lacerations (less extension to the anorectal complex) as a result of using the bPST [10].

### Strengths and limitations

Our study has several strengths which support the generalizability of its findings. This is the first study where the effect of bPST is studied in a low-middle-income country. The animated e-learning video is a new training tool that has not been tested in clinical setting before but it was evaluated by experts and generalist doctors and midwives in a previous study and found suitable for teaching purposes [24]. We have included only women examined by doctors who were well-trained in the assessment of obstetric perineal trauma. We believe that the assessment by the same personnel, who were independent to the actual birth, before and after the interventions mitigated bias related to variations in the diagnostic skills and clinical experience of the examiners. In addition, women included in this study were not examined at fixed times but randomly based on when the assessors showed up which reduced the risk of selection bias.

However, we also recognize that the study has some limitations. Firstly, preliminary power sample calculations were conducted based on previously reported international estimates since there were no previous accurate data of OASIS incidence in Palestine. However, the OASIS incidence observed during phase-1 was much higher than estimated and hence the power sample size calculations were reviewed based on the collected baseline incidence. Also, the statistical calculation was not stratified by parity and hence this might have masked significant variations before and after the interventions. Secondly, there were missing data in some variables (BMI and duration of second stage of labor). However, the missing data were random and were not considered to affect the studied exposure-outcome associations. Finally, this was designed as a pragmatic study and hence, we did not collect information about whether bPST was used during the birth or not. It is highly possible that some of the women, examined after the interventions, did not have bPST at birth which might have underestimated the impact of the training methodology.

## Conclusions

Blended learning methodology for bPST training was most effective in reducing OASIS incidence. However, the animated video is considered a promising independent training tool if the combined method is not feasible. Despite the challenging conditions in our setting, the animated video has independently contributed to the reduction in OASIS incidence. Therefore, we believe that such modality is a useful alternative particularly in limited resource healthcare settings. Integration of both training methods in the curriculum of doctors’ and midwives’ training programs under supervision of local trainers, has been set as a future strategy to sustain the impact of the quality improvement intervention.

## Additional file

Additional file 1: animation video.mp4. Length: 4 min and 43 s, size: 18.5 MB. Copyright holder: Oslo University Hospital 2015 ©. Permission to use the video was granted as the animation was designed by the authors KL and ÅV to be used in this study only. The copyright was registered under the name of the funding facilitator. (MP4 18988 kb)

## Abbreviations

bPST: Bimanual perineum support technique
OASIS: Obstetric anal sphincter injuries
